# Synthesis, structural and luminescent properties of Mn-doped calcium pyrophosphate (Ca_2_P_2_O_7_) polymorphs

**DOI:** 10.1038/s41598-022-11337-y

**Published:** 2022-05-03

**Authors:** Diana Griesiute, Edita Garskaite, Andris Antuzevics, Vytautas Klimavicius, Vytautas Balevicius, Aleksej Zarkov, Arturas Katelnikovas, Dick Sandberg, Aivaras Kareiva

**Affiliations:** 1 Institute of Chemistry, Vilnius University, Naugarduko 24, 03225 Vilnius, Lithuania; 2 Wood Science and Engineering, Department of Engineering Sciences and Mathematics, Luleå University of Technology, Forskargatan 1, 931 87 Skellefteå, Sweden; 3 Institute of Solid State Physics, University of Latvia, Kengaraga 8, Riga, 1063 Latvia; 4 Institute of Chemical Physics, Vilnius University, Sauletekio 3, 10257 Vilnius, Lithuania

**Keywords:** Chemistry, Materials science

## Abstract

In the present work, three different Mn^2+^-doped calcium pyrophosphate (CPP, Ca_2_P_2_O_7_) polymorphs were synthesized by wet co-precipitation method followed by annealing at different temperatures. The crystal structure and purity were studied by powder X-ray diffraction (XRD), Fourier-transform infrared (FTIR), solid-state nuclear magnetic resonance (SS-NMR), and electron paramagnetic resonance (EPR) spectroscopies. Scanning electron microscopy (SEM) was used to investigate the morphological features of the synthesized products. Optical properties were investigated using photoluminescence measurements. Excitation spectra, emission spectra, and photoluminescence decay curves of the samples were studied. All Mn-doped polymorphs exhibited a broadband emission ranging from approximately 500 to 730 nm. The emission maximum was host-dependent and centered at around 580, 570, and 595 nm for γ-, β-, and α-CPP, respectively.

## Introduction

Calcium phosphates (CPs) are the family of materials, widely used in different areas such as medicine and bone regeneration^1^, catalysis^2^, sensors^3^, removal of heavy metals from water^4^, as host matrices for the development of optical materials^5^, etc. Whereas in medicine, mainly calcium orthophosphates such as hydroxyapatite (HA, Ca_10_(PO_4_)_6_(OH)_2_)^6^, tricalcium phosphate (TCP, Ca_3_(PO_4_)_2_)^7^, or amorphous calcium phosphate (ACP) are used^8^, some recent studies on biomaterials showed the potential of the use of calcium pyrophosphate (CPP, Ca_2_P_2_O_7_) for biomedical applications. Filippov et al.^9^ fabricated macroporous β-CPP ceramics and, after the estimation of mechanical and biological properties in vitro, the fabricated material was suggested for potential use in osteoplastics. Anastasiou et al.^10^ used β-CPP with porous microstructure for drug loading and investigated its antibacterial properties against *E. coli* and *S. aureus*; it was concluded that β-CPP could be used for treating periodontitis or peri-implantitis. Different β-CPP containing composite materials were investigated as well. Hu et al.^11^ utilized β-CPP for the fabrication of a new Ti-13Nb-13Zr/β-CPP composite and investigated its mechanical and biological properties. Higher surface bioactivity as compared to the pure Ti-13Nb-13Zr alloy was demonstrated. The addition of β-CPP improved the elastic modulus, the thermal stability, and the flexural strength of poly(methyl methacrylate) resin used for interim fixed prostheses^12^. A collagen sponge reinforced with chitosan/CPP nanoflowers was designed by Yan et al.^13^ and used for hemostasis. It was shown that the composite could be biodegraded entirely in 3 weeks, which is suitable for post-operative treatment and peritoneal adhesion prevention.

One of the strategies for modifying CPs considers partial substitution of Ca^2+^ by other biocompatible ions with specific properties^14^. Modification of CPs with magnetic or optically active ions can be used for bio-imaging purposes^15^, hyperthermia cancer treatment^16^, or monitoring phase transitions in CPs *in situ*^17^. This approach was also employed to tune the properties of CPP. For instance, Kim et al.^18^ investigated the influence of Mg and Sr additives on the densification and biocompatibility of β-CPP ceramics. The presence of Mg and Sr ions considerably increased the bulk density of the ceramics. Moreover, co-substituted ceramics exhibited the best cell proliferation rate and cell affinity. Fe-doped β-CPP was suggested as a potential biomaterial for enamel restoration since cytotoxicity and genotoxicity tests revealed that β-CPP is cytocompatible and suitable in dental applications^19^.

Manganese is an essential element in the human body that plays a vital role in many biological processes, including bone development. Previously biological properties of different Mn-substituted CPs were investigated, and it was shown that the presence of Mn^2+^ ions in the CP matrix provides superior biological performance^20–22^. However, high concentrations of Mn^2+^ result in toxicity of the materials; therefore, Mn content should be limited to a relatively low level^23,24^.

Various phosphates are also widely used as matrices for the preparation of optical materials^25–27^. Mn^2+^ ions are known to be optically active and, depending on the host material and coordination, can possess emission in a broad spectral region from green to red^28^. Luminescent properties of Mn^2+^ ions were previously investigated in other CPs such as HA, α- and β-TCP. All studied materials exhibited broadband emission in the red spectral region; however, the position of emission maximum depended on the crystal structure of particular material^29,30^. Moreover, due to their paramagnetic nature, Mn^2+^ ions can be used as probes, which allow investigation of structural properties of materials by some powerful techniques such as electron paramagnetic resonance^31^.

Although CPP has three different polymorphs, most of the works focus exclusively on β-CPP, whereas two others are quite poorly investigated. Studies on α-CPP are most often related to the preparation of optical materials by substitution of Ca^2+^ ions by lanthanides^32,33^; nevertheless, β-CPP besides of the use in medicine also finds an application in the preparation of phosphors^34,35^. The least studied polymorph is γ-CPP. This work demonstrates a simple and time-efficient way of preparing Mn^2+^-substituted brushite with subsequent conversion to γ-, β-, and α-CPP. Structural, optical, and morphological properties of synthesized materials were investigated in detail.

## Experimental

### Synthesis

The synthesis of Mn-doped CPP polymorphs was performed by wet co-precipitation method followed by annealing at different temperatures. The as-prepared precipitates were synthesized by a slightly modified previously reported procedure^36^. Briefly, calcium nitrate tetrahydrate (Ca(NO_3_)_2_ · 4H_2_O, Carl Roth, > 99%), manganese(II) nitrate tetrahydrate (Mn(NO_3_)_2_ ∙ 4H_2_O, 98%, Alfa Aesar) and diammonium hydrogen phosphate ((NH_4_)_2_HPO_4_, Carl Roth, > 98%) were used as starting materials. Firstly, 0.8 M Ca^2+^ and Mn^2+^ nitrates solution was prepared by dissolving metal salts in deionized water. Mn-substitution level was chosen as 1 mol% with respect to Ca^2+^ ions. (NH_4_)_2_HPO_4_ solution of the same concentration was prepared in a separate beaker. Next, the solution containing phosphate ions was rapidly added to the solution of metal ions under constant mixing on a magnetic stirrer, resulting in precipitates’ formation. The obtained precipitates were aged in the reaction mixture for 5 min while stirring, afterward filtered, washed with deionized water, and dried in an oven at 50 °C overnight. The as-prepared powders were annealed for 5 h at 700 °C and 1000 °C for the synthesis of γ-CPP and β-CPP, respectively. For the preparation of α-CPP, a shorter annealing procedure at 1200 °C was employed (10 min) to minimize evaporation of phosphate species^37^. After the annealing procedure, the furnace was cooled down naturally.

### Characterization

Powder X-ray diffraction data were collected using Ni-filtered Cu Kα radiation on a Rigaku MiniFlex II diffractometer working in Bragg–Brentano (θ/2θ) geometry. The data were collected within a 2θ angle range from 10° to 60° at a step width of 0.02° with a scanning speed of 1°/min. Infrared (FTIR) spectra were obtained in the range of 4000 − 400 cm^−1^ employing Bruker ALPHA ATR spectrometer. Morphology of the synthesized powders was characterized by scanning electron microscopy performed with a Hitachi SU-70 field-emission scanning electron microscope (FE-SEM). Electron paramagnetic resonance (EPR) spectra were recorded with a Bruker ELEXSYS-II E500 CW-EPR spectrometer at X (9.838 GHz) and Q (33.92 GHz) microwave frequency bands. Spectra acquisition parameters for measurements at both frequency bands were: room temperature, 10 mW microwave power, and 0.2 mT magnetic field modulation amplitude. EasySpin software^38^ was employed for EPR spectra simulations. Photoluminescence excitation (PLE) and photoluminescence emission (PL) spectra were measured on the Edinburgh Instruments FLS980 spectrometer equipped with double excitation and emission monochromators, 450 W Xe arc lamp, a cooled (− 20 °C) single-photon counting photomultiplier (Hamamatsu R928P), and a mirror optics for powder samples. The PL spectra were corrected by a correction file obtained from a tungsten incandescent lamp certified by NPL (National Physics Laboratory, UK). The excitation spectra were corrected by a reference detector. The PL decay curves were measured on the same Edinburgh Instruments FLS980 spectrometer. Xe µ-flash lamp µF920 was used as an excitation source. Solid-state NMR experiments were carried out at 9.4 T on a Bruker Avance III HD 400 NMR spectrometer operating at 400.2 and 162.0 MHz for ^1^H and ^31^P, respectively, using a 4 mm double resonance CP MAS probe and 4 mm zirconia rotors. The temperature was stabilized at 298 K. For ^31^P MAS measurements, a saturation recovery pulse sequence and 10 kHz MAS were employed. The saturation pulse train consisted of 20 π/2 pulses followed by 15–30 s (for Mn-doped samples) or 4000 s (for undoped samples) delays which were determined from spin–lattice relaxation measurements and were ≥ 5∙*T*_1_. The π/2 excitation pulse was equal to 2.5 μs, 8 and 16 scans were accumulated for undoped and Mn-doped samples, respectively, spinal64 decoupling sequence was applied during the acquisition of FID. All spectra were referenced to 85% H_3_PO_4_ using ADP (ammonium dihydrogen phosphate, NH_4_H_2_PO_4_) as an external standard (*δ* (^31^P) = 0.8 ppm).

## Results and discussion

**Figure 1:**
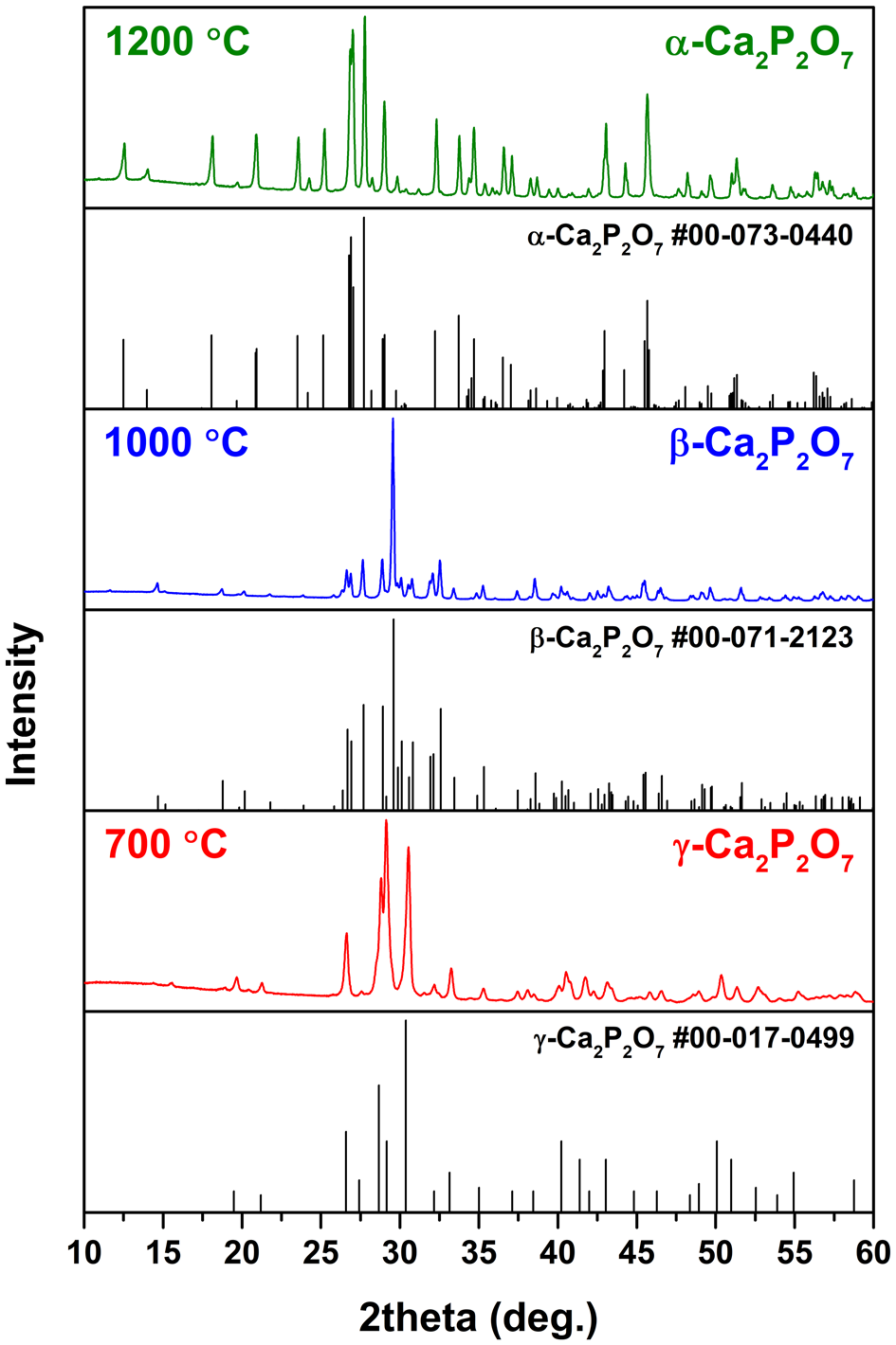
XRD patterns of Mn-doped CPP polymorphs.

The as-prepared precipitates were identified as brushite (CaHPO_4_∙2H_2_O), characterized by the same Ca-to-P ratio as CPP (1:1). This material is thermally unstable and decomposes upon heat treatment with the formation of CPP and monetite (CaHPO_4_) as an intermediate phase. The transformation from monetite to γ-CPP is observed at around 450 °C (Fig. S1). The XRD patterns of precipitates annealed at different temperatures are given in Fig. 1. It is evident that three different polymorphs were obtained depending on the annealing temperature. Due to the low crystallinity, the XRD pattern of powders annealed at 700 °C consists of broad overlapping diffraction peaks. Whereas the experimental pattern matches that of the standard XRD data (PDF #017-0499) in terms of the peak positions, there is a significant difference in the intensity, which suggests the anisotropic nature of particles. At the same time, the literature analysis revealed that among CPP polymorphs the low-temperature γ-CPP is the least investigated material. We could not find any comprehensive study on the structural properties of this polymorph. Moreover, we could not find the standard data with crystallographic details such as space group, atomic coordinates, etc., in the PDF-4 database either. It can be caused by the fact that upon heating, it transforms to β-CPP at a relatively low temperature, which does not allow to prepare a highly crystalline material. Another point is that γ-CPP obtained by thermal decomposition of CaHPO_4_∙2H_2_O tends to preserve the morphological features of the latter and forms plate-like particles, which result in preferred orientation in XRD patterns^39^. The XRD pattern of powders annealed at 1000 °C matches very well with standard XRD data of tetragonal Ca_2_P_2_O_7_ (PDF# 071-2123) with the P4_1_ space group (#76) in terms of the position of diffraction peaks. However, it also possesses the preferred orientation and is dominated by the most intense (008) peak. Finally, the XRD pattern of powders annealed at 1200 °C is in good agreement with XRD data of monoclinic Ca_2_P_2_O_7_ (PDF #073-0440) with the P2_1_/n space group (#14). The negligible amount of α-TCP was observed as a neighboring phase, resulting from selective evaporation of phosphate species previously reported in the literature^37,40^. Due to the lack of crystallographic data for γ-CPP, Rietveld refinement was employed to calculate lattice parameters only for β- and α-CPP (Figs. S2 and S3). The results are summarized in Table 1. The calculated parameters are slightly smaller than those of the standards, which is explained by the presence of Mn^2+^ ions in the crystal structure, which have a smaller ionic radius than Ca^2+^^41^.

**Table 1:** Lattice parameters of Mn-doped β-CPP and α-CPP.

Sample	a, Å	b, Å	c, Å	V, Å^3^
β-CPP	6.681	6.681	24.126	1076.8
α-CPP	12.644	8.522	5.312	572.3

**Figure 2:**
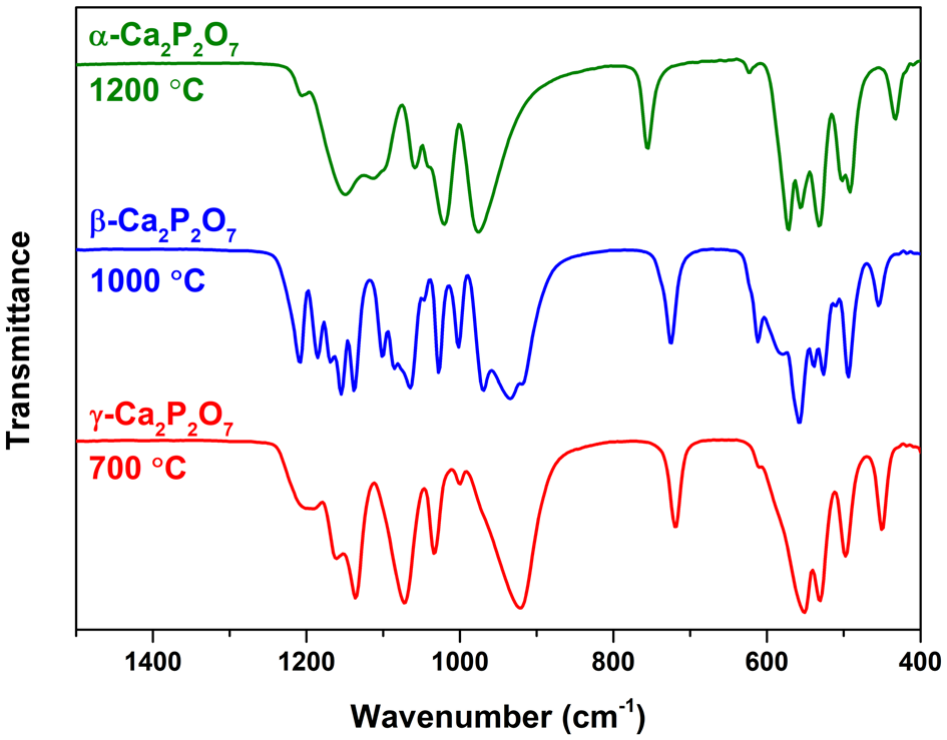
FTIR spectra of Mn-doped CPP polymorphs.

The FTIR spectra of Mn-doped CPP polymorphs are depicted in Fig. 2. Although all the spectra look complicated, the difference between the three materials is evident. The detailed analysis of CPP polymorphs by vibrational spectroscopy was previously reported elsewhere^42–44^. The positions of the absorption bands are in good agreement with the reported data.

**Figure 3:**
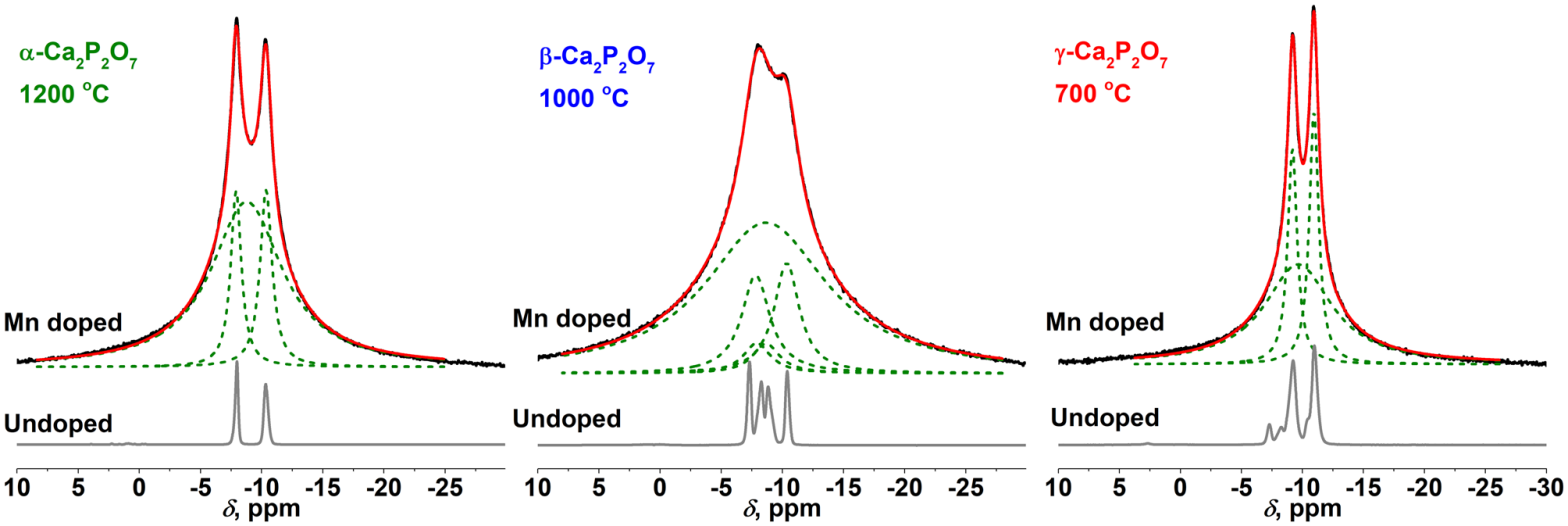
^31^P MAS spectra of Mn-doped and undoped CPP polymorphs.

The obtained ^31^P MAS spectra for Mn-doped and undoped CPP polymorphs (Fig. 3) show apparent differences, which are in line with the literature^45,46^. The ^31^P MAS spectrum obtained for the Mn-doped γ-CPP polymorph consists of two sharper peaks at − 9.2 and − 10.9 ppm, which are also seen in the spectrum obtained for the undoped γ-CPP and one broad peak at − 9.6 ppm. This peak is attributed to the ^31^P moieties closer to the paramagnetic Mn^2+^ ion, which results in spectral broadening. The peaks at − 7.3, − 8.3, − 10.4 ppm seen in the ^31^P MAS spectrum obtained for the undoped sample are not resolved for the Mn-doped γ CPP. These peaks are attributed to β-CPP polymorph, which is present in the sample as an impurity phase; the Mn-doping prevents the formation of this secondary phase. The ^31^P MAS spectrum obtained for Mn-doped β-CPP polymorph features all peaks observed for the undoped corresponding polymorph, namely at − 7.3, − 8.3, − 8.8, − 10.4 ppm. In addition, a broad peak at − 8.7 ppm is attributed to the ^31^P moieties in closer vicinity to the Mn^2+^ ion, which results in paramagnetic broadening. The ^31^P MAS spectrum obtained for the Mn-doped α-CPP polymorph consists of two sharper peaks at − 8.0 and − 10.3 ppm, which are also present in the spectrum obtained for undoped α-CPP. Similarly, as for other polymorphs, a broad peak at − 8.8 ppm is attributed to the ^31^P moieties closer to the Mn^2+^ ion.

**Figure 4:**
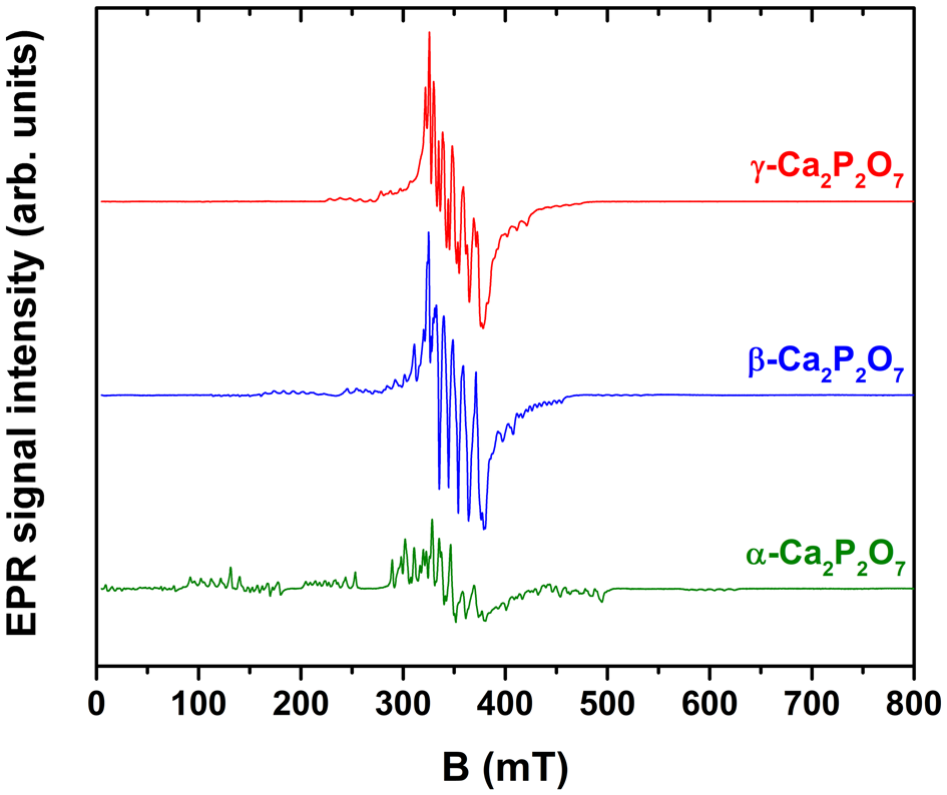
Experimental X-band EPR spectra of calcium phosphate samples.

The results of X-band EPR measurements are presented in Fig. 4. Spectra of all CPP polymorphs extend over a broad field range and exhibit a complicated pattern of resonances characteristic to Mn^2+^. Signal intensities are comparable for different samples, indicating no significant deviations in the concentration of the paramagnetic ions. EPR spectra of CPP:Mn^2+^ polymorphs were previously reported in^47^; however, a detailed analysis was not provided.

Qualitative similarities are expected in the EPR spectra of Mn^2+^-doped CPs^23,29,30,47–51^. Mn^2+^ is an electron spin $S$ = 5/2 system, which leads to six possible quantum states *M*_*S*_ = -5/2, -3/2, …, + 5/2. Due to zero field splitting (ZFS) of the ground state, the five allowed transitions (Δ*M*_*S*_ =  ± 1) occur at different magnetic field values. If the magnitude of ZFS is large, the spectrum extends over a broad field range, and forbidden transitions (Δ*M*_*S*_ =  ± 2) can occasionally be resolved. In addition, each of the ZFS transitions is split into six components due to hyperfine (HF) interaction between $S$ and nuclear spin $I$ = 5/2 of ^55^Mn. In polycrystalline CPs, HF structure is commonly resolved only for the *M*_*S*_ =  + 1/2 ↔ − 1/2 transition (≈ 350 mT in Fig. 4)^23,29,30,48–51^.

Several conclusions can be inferred from an inspection of the spectra in Fig. 4. First, HF structure is discernible for all samples and all ZFS transitions. It is associated with relatively narrow distributions of ZFS parameter values, implying minor site-to-site variations in the local structure of Mn^2+^ ions. It should be noted that a contribution of Mn^4+^ signals to room temperature EPR spectra cannot be excluded; however, no signals associated with Mn^3+^ are expected under our experimental settings^52–54^. Secondly, the magnetic field range of the observed resonances and, hence, the magnitude of ZFS increases in the order of γ → β → α. Additional measurements at the Q-band and simulations of the spectra acquired at both frequency bands were performed to quantify this effect. The following spin-Hamiltonian (SH) was used^55^:

1 $$H=g{\mu }_{B}BS+D\left[{S}_{z}^{2}-S\left(S+1\right)/3\right]+E\left({S}_{x}^{2}-{S}_{y}^{2}\right)+ASI$$

where $g$ is the *g-*factor; ${\mu }_{B}$ – the Bohr magneton; $B$ – external magnetic field; ${S}_{x,y,z}$ – projections of the spin operator $S$; $D$ and $E$ – ZFS parameters; and $A$ – HF coupling tensor. Higher order ZFS terms were neglected to simplify simulations. All spectra were simulated as $S$ = 5/2 systems, interacting with a $I$ = 5/2 nucleus.

**Figure 5:**
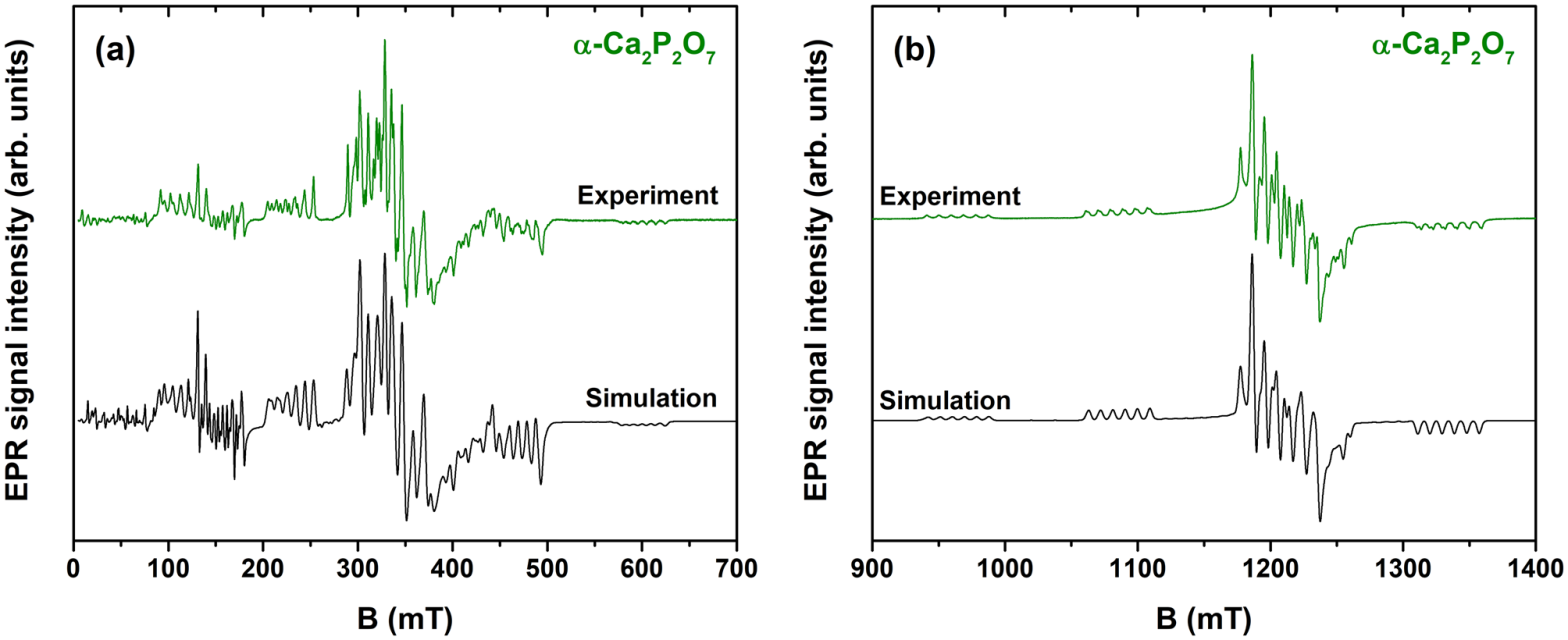
Simulations of X-band (**a**) and Q-band (**b**) EPR spectra of α-Ca_2_P_2_O_7_.

Simulation results for the α-CPP sample are demonstrated in Fig. 5, and an analogous analysis for the γ- and β-CPP phases is provided as supplementary information (Fig. S4 and Fig. S5). The fitted parameter values with the evaluated uncertainties are summarized in Table 2.

**Table 2:** A summary of EPR simulation parameters.

Sample	$g$	$A$, MHz	$D$, MHz	$E$, MHz
γ-Ca_2_P_2_O_7_	2.000 ± 0.001	265 ± 5	690 ± 30	230 ± 50
β-Ca_2_P_2_O_7_	2.000 ± 0.001	263 ± 5	1200 ± 50	340 ± 50
α-Ca_2_P_2_O_7_	2.000 ± 0.001	259 ± 2	1755 ± 20	578 ± 20

Overall, an excellent fit to the experimental data is achieved for the α-CPP sample. The agreement of the simulated and experimental spectra for the γ- and β-CPP samples is reasonable – positions of the major experimental features are accounted for. However, the line broadenings for the non-central ZFS transitions could not be reproduced. More detailed linewidth broadening models and inclusion of higher order terms in the SH (Eq. 1) could improve the fit. It is also possible that Mn^2+^ ions incorporate in more than one crystallographic site of the γ- and β-CPP phases or there is also a contribution from Mn^4+^, and the EPR spectrum should have been simulated as a superposition of several signals. Nevertheless, a comparison of the SH parameters in the investigated CPP polymorphs can be carried out. The determined values of $g$ and $A$ are similar and do not deviate from the values reported for Mn^2+^ centers in other CPs^23,29,30,48–51^. The values of the ZFS parameters $D$ and $E$, on the other hand, differ significantly. ZFS parameters reflect the coordination symmetry of the paramagnetic center and bonding with the surrounding ligands^56^, which can be used to analyze the site occupancy of the dopant ions.

Due to similarities in ionic radii and oxidation state, Mn^2+^  → Ca^2+^ substitution is expected in CPs^23,29,30,47–51^. There are two cation sites in α-CPP, both coordinated by eight oxygen ions with average Ca-O bond lengths of 2.51 and 2.54 Å^57^. However, only one Mn^2+^ center is evidenced by EPR spectra simulations (Fig. 5). It could be explained either by the preferential incorporation of Mn^2+^ ions in a single cationic site of the α-CPP structure or by the inability to distinguish the two sites due to similarities in the local structure. On the other hand, the results of Rietveld refinement indicated that Mn^2+^ ions occupy both Ca(1) and Ca(2) sites. Analysis of the EPR spectra of γ-CPP (Fig. S4) also suggests that Mn^2+^ ions occupy a single crystallographic site. All experimentally observed features have been accounted for; however, the fit for the line broadenings could be improved. In β-CPP, there are four distinct Ca sites: eight-fold coordinated Ca(1), nine-fold coordinated Ca(2), and seven-fold coordinated Ca(3) and Ca(4)^58^. Simulations (Fig. S5) do not provide an ideal fit to the experimental spectra in a model of a single Mn^2+^ center, particularly in 240–300 mT and 400–460 mT regions of the X-band data. Therefore, it is likely that there are at least two different Mn^2+^ centers contributing to the EPR spectrum. Similar conclusions have been inferred from the analysis of optical properties of Bi^2+^-^59^ and Eu^3+^-doped^60^ β-CPP. More precisely, the Ca(1) and Ca(2) sites have been preferentially occupied by the dopant ions^59^. It is worth to note that both Bi^2+^ and Eu^3+^ are significantly larger in size compared to Mn^2+^^41^. The results of Rietveld refinement suggest that small Mn^2+^ ions preferentially occupy Ca(4) site, which has lower coordination number. On the other hand, the content of Mn in analyzed material is relatively low, which explains the contradiction of two techniques.

To summarize, the EPR results demonstrate that Mn^2+^ ions are excellent probes to monitor phase transitions of calcium pyrophosphates. ZFS parameters, which determine the fine structure of the EPR spectrum, differ substantially in the different CPP polymorphs, revealing variations in the local environment of the paramagnetic ions.

**Figure 6:**
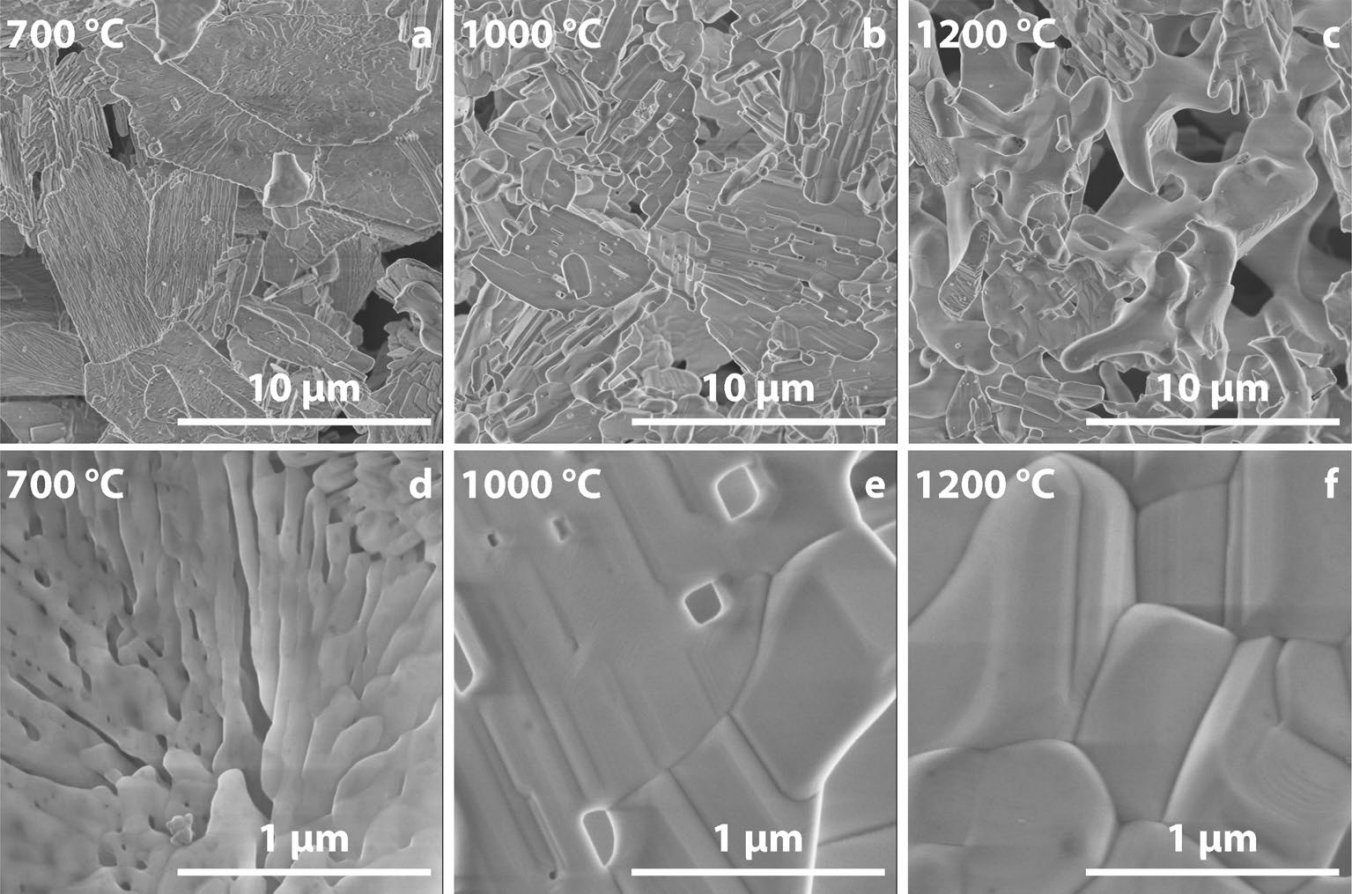
SEM images of Mn-doped γ-CPP (**a**,**d**), β-CPP (**b**,**e**), and α-CPP (**c**,**f**) polymorphs synthesized at 700°, 1000°, and 1200 °C, respectively.

Figure 6 shows SEM images of Mn-doped CPP polymorphs prepared at different temperatures. It is seen that γ-CPP (Fig. 6a,d) consists of plate-like particles of micrometric sizes, which is in good agreement with the preferred orientation observed in the XRD patterns (Fig. 1). A closer look allows us to see the porous structure of the particles. The oriented morphology of the particles was preserved after annealing at 1000 °C and phase transformation to β-CPP, which is seen in Fig. 6b,e. The porosity of the particles noticeably decreased due to the sintering; however, some pores still can be seen. Finally, α-CPP powders possessed different morphological features (Fig. 6c,f). Due to the higher degree of sintering, the well-defined plate-like shape of the particles was lost. Nevertheless, some sub-micrometric grains can still be observed.

**Figure 7:**
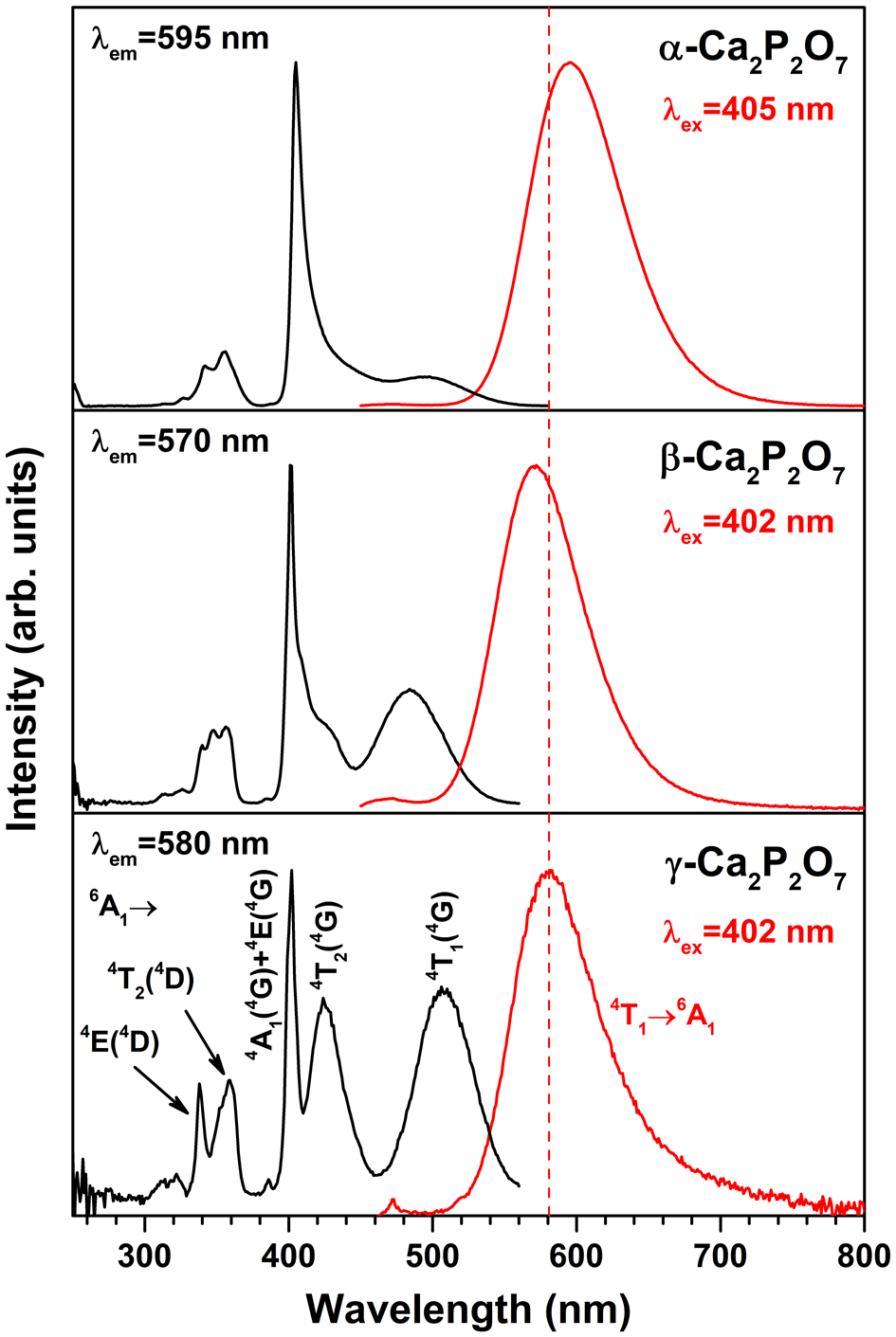
Excitation and emission spectra of Mn-doped CPP polymorphs.

Excitation and emission spectra of the synthesized Mn-doped CPP polymorphs are given in Fig. 7. The emission spectra of all polymorphs consist of one broad band in the orange spectral region, indicating that Mn^2+^ occupies lattice sites generating strong crystal-fields^61^. Emission originates from the Mn^2+ 4^T_1_ → ^6^A_1_ optical transition, and its maximum is slightly dependent on the CPP polymorph, i.e., 595 nm for α-CPP, 570 nm for β-CPP, and 580 nm for γ-CPP. Besides, the full width at half maximum (FWHM) also slightly increases when emission shifts to the longer wavelengths, 71 nm for β-CPP, 74 nm for γ-CPP, and 76 nm for α-CPP polymorph. The emission intensity decreased in the sequence α-CPP > β-CPP > γ-CPP, which can be related to the annealing temperature applied for the preparation of particular polymorph and different degree of crystallinity of analyzed materials.

Excitation spectra of the synthesized compounds, however, are more complex. They consist of both broad and narrow excitation bands. The intensive and narrow excitation band at ca. 400 nm is attributed to the practically coinciding ^6^A_1_ → ^4^A_1_ and ^6^A_1_ → ^4^E transitions of Mn^2+^. Since both ^4^A_1_ and ^4^E levels run parallel with the ground level (^6^A_1_), their energy stays the same regardless of the weak or strong crystal-field, and a very narrow band is observed in the excitation spectra. There are also several broad bands in the excitation spectra that are assigned to the ^6^A_1_ → ^4^T_1_(^4^G) (ca. 500 nm), ^6^A_1_ → ^4^T_2_(^4^G) (ca. 420 nm), ^6^A_1_ → ^4^T_2_(^4^D) (ca. 360 nm) optical transitions of Mn^2+^. All of these mentioned excited levels have a slope relative to the ground level (i.e., the *x*-axis) in the Tanabe-Sugano diagram^62^; therefore, the variation in the crystal-field energy will result in broad excitation bands. The excitation spectrum of γ-CPP:Mn^2+^ also contains one more narrow excitation band attributed to the ^6^A_1_ → ^4^E(^4^D) optical transition of Mn^2+^.

**Figure 8:**
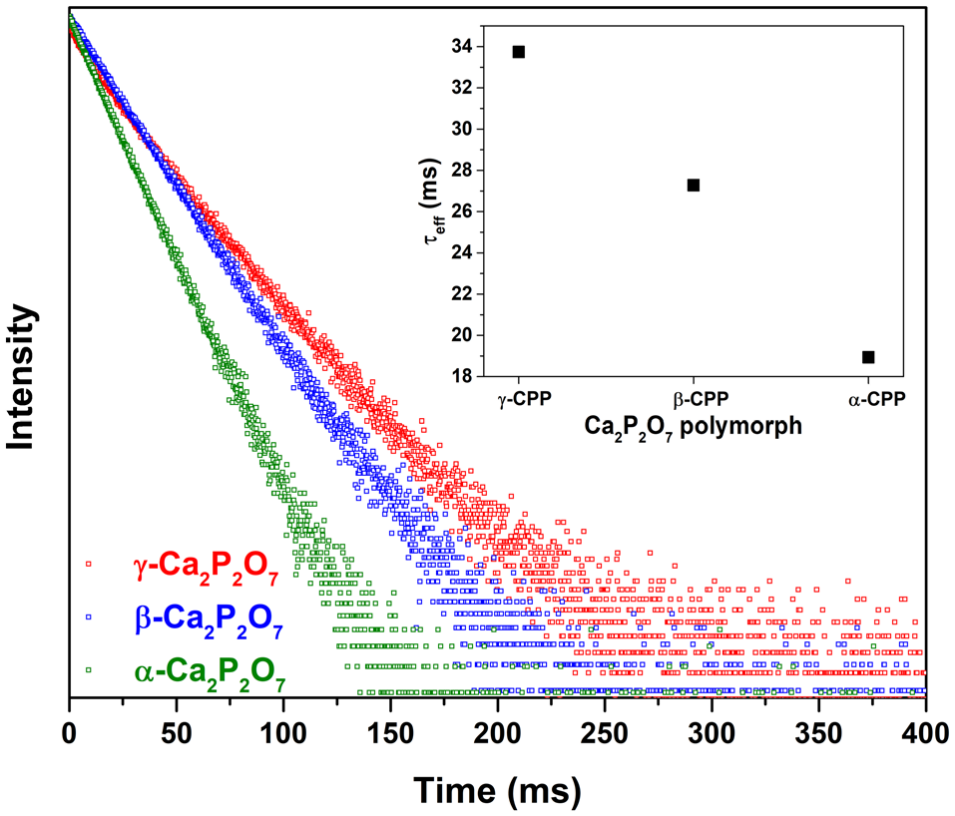
PL decay curves of Mn-doped CPP polymorphs. Inset: calculated effective PL lifetime values.

The photoluminescence decay curves of Mn-doped CPP polymorphs are depicted in Fig. 8, and the calculated PL lifetime values (τ_eff_) are provided in the inset of the exact figure. Mn^2+^ optical transitions are both parity and spin forbidden; therefore, the PL decay time is in the order of milliseconds. The slowest Mn^2+^ photoluminescence was observed in γ-polymorph (τ_eff_ ≈ 33.5 ms), whereas the fastest was in α-polymorph (τ_eff_ ≈ 19 ms).

## Conclusions

Three different Mn^2+^-doped Ca_2_P_2_O_7_ polymorphs were successfully synthesized by a simple wet co-precipitation method followed by annealing at different temperatures. Rietveld refinement indicated high purity of the synthesized polymorphs and lattice parameters close to standard materials. EPR measurements demonstrated that Mn^2+^ ions are excellent probes to monitor phase transitions between calcium pyrophosphates. ZFS parameters, which determine the fine structure of the EPR spectrum, differ substantially in different CPP polymorphs, revealing variations in the local environment of the paramagnetic ions. Morphological features were different for all polymorphs varying from porous plate-like particles to sintered monoliths of undefined shape. Excitation spectra, emission spectra, and photoluminescence decay curves of the samples were studied and indicated that all Mn-doped polymorphs exhibited a broadband emission from approximately 500 to 730 nm. The emission maximum was host-dependent and was centered at around 580, 570, and 595 nm for γ-, β-, and α-CPP, respectively. The calculated PL decay time was in the order of milliseconds. The slowest Mn^2+^ photoluminescence was observed in γ-polymorph (τ_eff_ ≈ 33.5 ms), whereas the fastest was in α-polymorph (τ_eff_ ≈ 19 ms).

## Supplementary Information

Supplementary Information.
