# Supplementary material for: Synthesis, structural and luminescent properties of Mn-doped calcium pyrophosphate (Ca2P2O7) polymorphs
Source: Sci Rep. 2022 May 3;12:7116. doi: 10.1038/s41598-022-11337-y (PMC9065112; doi:10.1038/s41598-022-11337-y)
Supplement: Supplementary file 1 — Supplementary Information. [file 41598_2022_11337_MOESM1_ESM.docx]

Supplementary Information

Synthesis, structural and luminescent properties of Mn-doped calcium pyrophosphate (Ca_2_P_2_O_7_) polymorphs

Diana Griesiute^1^, Edita Garskaite^2*^, Andris Antuzevics^3^, Vytautas Klimavicius^4^, Vytautas Balevicius^4^, Aleksej Zarkov^1^, Arturas Katelnikovas^1^, Dick Sandberg^2^, Aivaras Kareiva^1,*^

*^1^Institute of Chemistry, Vilnius University, Naugarduko 24, LT-03225 Vilnius, Lithuania*

*^2^Wood Science and Engineering, Department of Engineering Sciences and Mathematics, Luleå University of Technology, Forskargatan 1, SE-931 87 Skellefteå, Sweden*

*^3^Institute of Solid State Physics, University of Latvia, Kengaraga 8, LV-1063 Riga, Latvia*

*^4^Institute of Chemical Physics, Vilnius University, Sauletekio 3, LT-10257, Vilnius, Lithuania*

**Corresponding author: E-mail:* [*edita.garskaite@ltu.se*](mailto:edita.garskaite@ltu.se)*,* [*aivaras.kareiva@chgf.vu.lt*](mailto:aivaras.kareiva@chgf.vu.lt)*,*


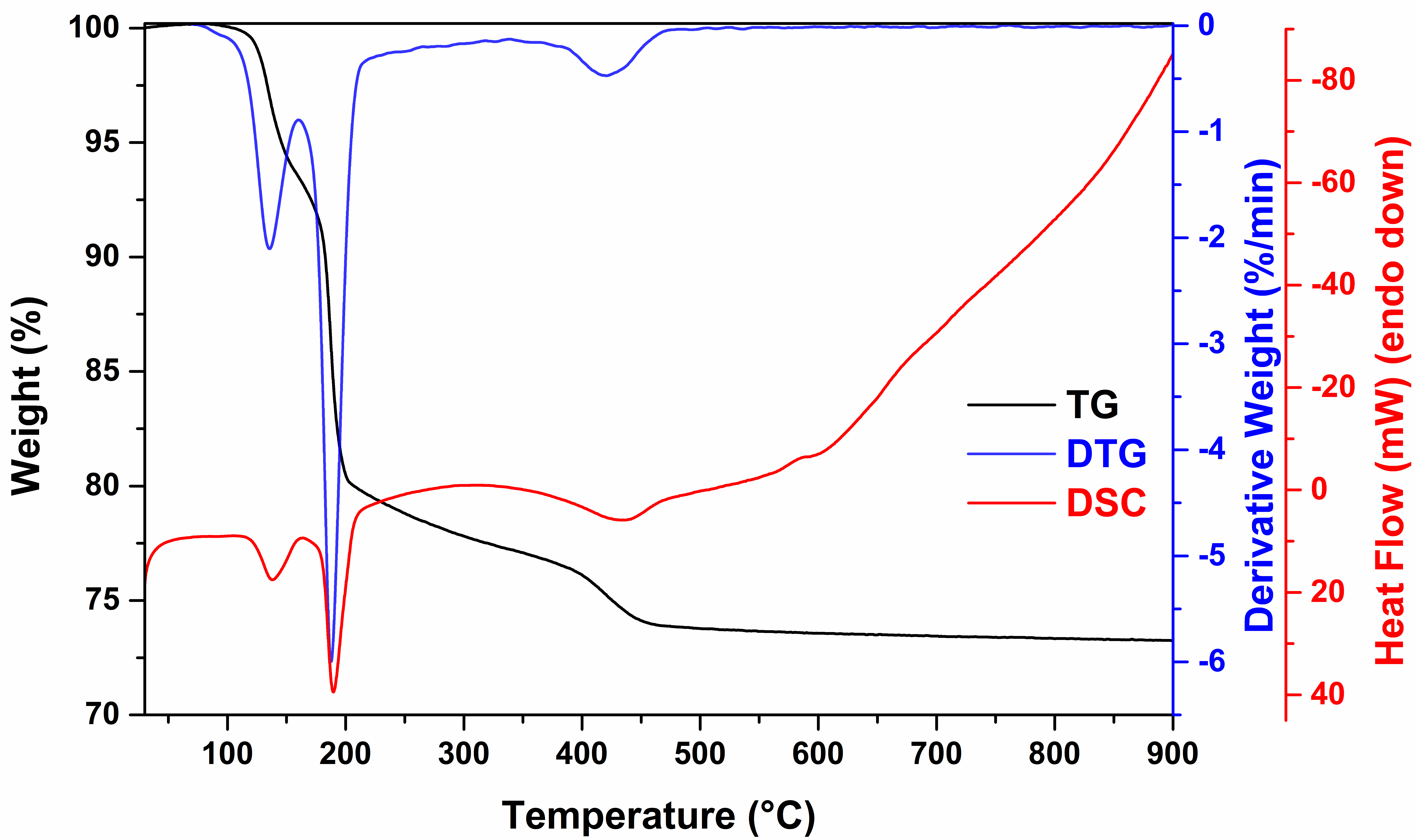


Fig. S1. TG/DTG/DSC curves of as-synthesized brushite precipitates.


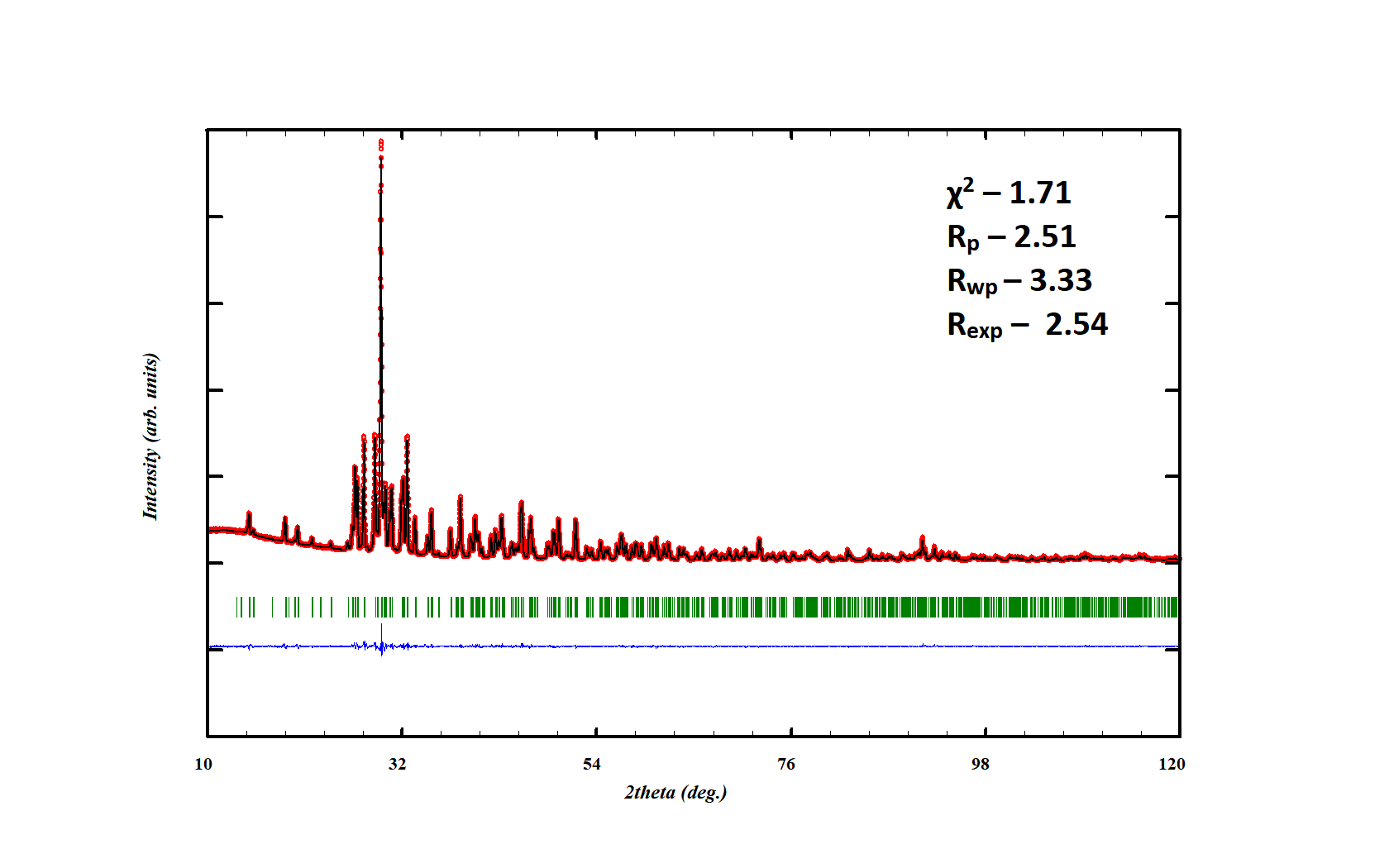


Fig. S2. Rietveld refinement of the XRD data obtained for β‑CPP refined in space group P4_1_. The red circle symbols and the black solid line represent the experimental and calculated intensities, respectively, and the blue line below is the difference between them. The green tick marks indicate the positions of the Bragg peaks.


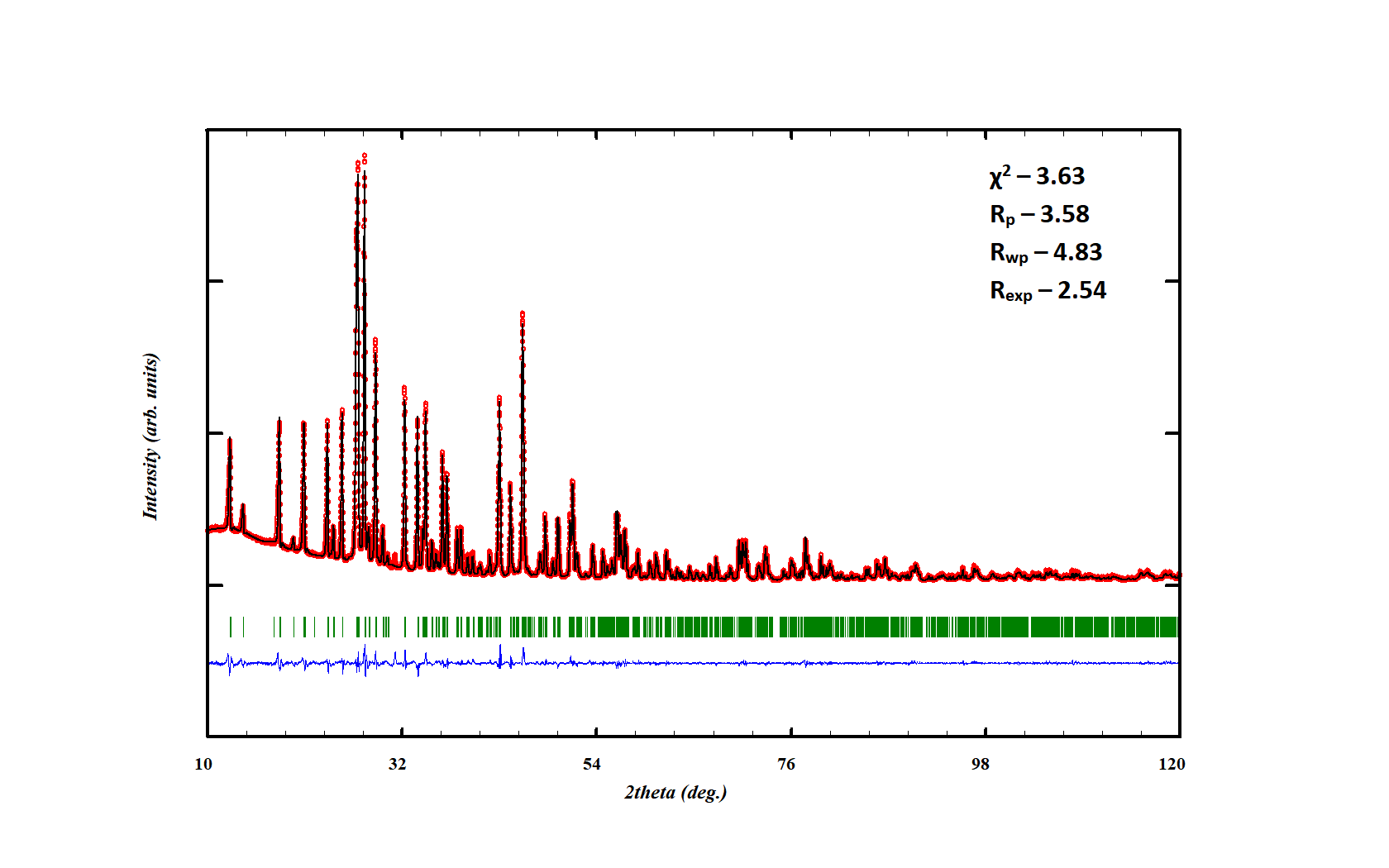


Fig. S3. Rietveld refinement of the XRD data obtained for α‑CPP refined in space group P2_1_/n. The red circle symbols and the black solid line represent the experimental and calculated intensities, respectively, and the blue line below is the difference between them. The green tick marks indicate the positions of the Bragg peaks.





Fig. S4. Simulations of X-band (a) and Q-band (b) EPR spectra of γ-Ca_2_P_2_O_7_.


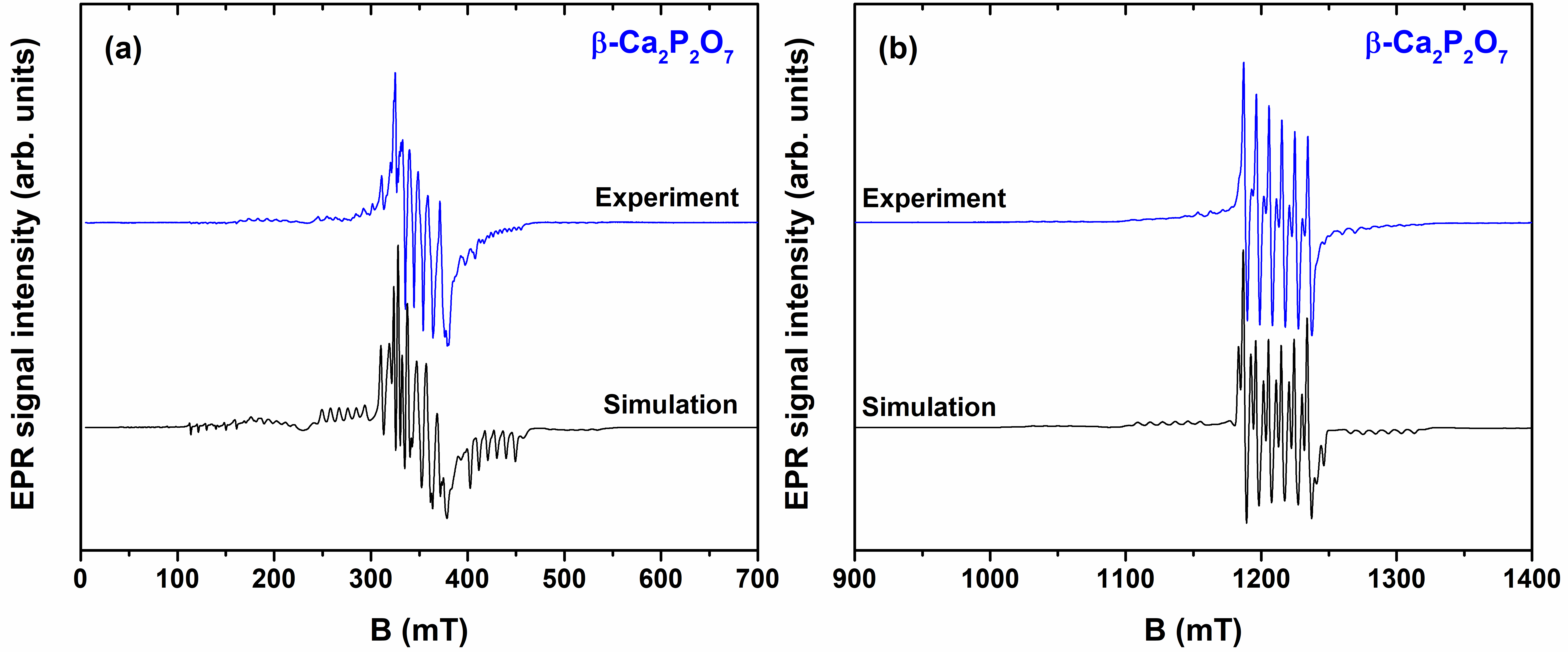


Fig. S5. Simulations of X-band (a) and Q-band (b) EPR spectra of β-Ca_2_P_2_O_7_.
